# A drug recommender system for the treatment of hypertension

**DOI:** 10.1186/s12911-023-02170-y

**Published:** 2023-05-09

**Authors:** Arthur Mai, Karen Voigt, Jeannine Schübel, Felix Gräßer

**Affiliations:** 1 Faculty of Medicine Carl Gustav Carus, Department of General Practice, TU Dresden, Dresden, Germany; 2 Institute of Biomedical Engineering, TU Dresden, Dresden, Germany

**Keywords:** Clinical Decision Support System, CDSS, Health Recommender System, Drug Recommender System, Hypertension, Real World Evidence, Shared decision-making

## Abstract

**Background:**

One third (20% to 30%) of patients suffering from hypertension show increased blood pressure resistant to treatment. This resistance often has multifactorial causes, like therapeutic inertia and inappropriate medication but also poor patient adherence. Evidence-based guidelines aim to support appropriate health care decisions. However, (i) research and appraisal of clinical guidelines is often not practicable in daily routine care and (ii) guidelines alone are often insufficient to make suitable and personalized treatment decisions. Shared decision-making (SDM) can significantly improve patient adherence, but is also difficult to implement in routine care due to time constraints.

**Methods:**

Clinical Decision Support Systems (CDSSs), designed to support clinical decision-making by providing explainable and personalized treatment recommendations, are expected to remedy the aforementioned issues. In this work we describe a digital recommendation system for the pharmaceutical treatment of hypertension and compare its recommendations with clinical experts. The proposed therapy recommender algorithm combines external evidence (knowledge-based) – derived from clinical guidelines and drugs’ professional information – with information stored in routine care data (data-based) – derived from 298 medical records and 900 doctor-patient contacts from 7 general practitioners practices. The developed Graphical User Interface (GUI) visualizes recommendations along with personalized treatment information and intents to support SDM. The CDSS was evaluated on 23 artificial test patients (case vignettes), by comparing its output with recommendations from five specialized physicians.

**Results:**

The results show that the proposed algorithm provides personalized treatment recommendations with large agreement with clinical experts. This is true for agreement with all experts (agree_all), with any expert (agree_any), and with the majority vote of all experts (agree_majority). The performance of a solely data-based approach can be additionally improved by applying evidence-based rules (external evidence). When comparing the achieved results (agree_all) with the inter-rater agreement among experts, the CDSS’s recommendations partly agree more often with the experts than the experts among each other.

**Conclusion:**

Overall, the feasibility and performance of medication recommendation systems for the treatment of hypertension could be shown. The major challenges when developing such a CDSS arise from (i) the availability of sufficient and appropriate training and evaluation data and (ii) the absence of standardized medical knowledge such as computerized guidelines. If these challenges are solved, such treatment recommender systems can support physicians with exploiting knowledge stored in routine care data, help to comply with the best available clinical evidence and increase the adherence of the patient by reducing site-effects and individualizing therapies.

## Introduction

Cardiovascular diseases are the most common causes of premature death in Northern America and Western Europe [1]. Hypertension is one of the most preventable risk factors for cardiovascular diseases and therefore a major public health issue. Hypertension is caused by multiple factors: genetic disposition and exogenous factors like health behavior and living conditions [1]. The prevalence of hypertension in Germany is around 30%, in the group of those aged >  = 65 as high as around 60% [1]. According to the guideline of the European Society of Cardiology (ESC) and to the guideline of the German College of General Practitioners and Family Physicians (DEGAM), primary hypertension is defined as systolic blood pressure permanently above 140 mmHg and/or diastolic blood pressure above 90 mmHg, respectively [2]. The treatment of hypertension consists of a stepwise therapy regime to reduce the total risk of cardiovascular diseases: (1) behavior changes aiming at a more healthy lifestyle and additionally, (2) if a total risk of cardiovascular diseases > 20%, a consequent medication therapy is necessary [2]. Antihypertensive drug therapy comprises five groups of substances which act on different systems and have proven to be particularly effective in combination [3]. Nevertheless, one third of patients show increased blood pressure resistance to treatment. This resistance often has multifactorial causes, such as therapeutic inertia and inappropriate medication but also poor patient adherence. Evidence-based information like medical guidelines or pharmaceutical journals, provide current evidence regarding treatment options and aim to support appropriate health care decisions [4]. Shared Decision-Making (SDM), which takes the individual patient preferences into account, can improve adherence to treatment decisions [5].

There is evidence that guidelines may improve patient outcomes, but there is also evidence that physicians’ adherence to guidelines may be optimized [4]. Time-consuming complex guideline or literature research is not practicable in daily routine care. Moreover, patients often differ from the study populations evidence is based on, e.g. are multimorbid. Hence, medical care cannot focus on just one disease entity but must target the whole patient with multimorbidity and individual treatment preferences (holistic approach). In routine care it is necessary to adapt guideline recommendations individually, taking patient characteristics such as age, weight, comorbidities, concomitant medications, and special living circumstances (such as pregnancy, breastfeeding) into account. Also SDM, which requires the communication of benefits and risks of all available pharmaceutical treatment options, is often challenging due to the variety of drugs and drug combinations.

We propose to face these complex challenges with a Clinical Decision Support System (CDSS) that provides personalized and evidence-based treatment recommendations taking comorbidities, comedication, patient characteristics and living conditions into account while supporting SDM.

A large variety of CDSSs is described in literature [6–10]. They can be categorized into knowledge- and non-knowledge-based, i.e. data-driven systems [6, 7]. Whereas knowledge-based systems typically rely on compiled rules, such as clinical guidelines or expert-knowledge, data-driven CDSSs apply machine learning or other statistical pattern recognition methods to automatically learn from past experiences stored in clinical data. Knowledge-based approaches have a high level of evidence, but are standardized on populations, less individualized and typically static, often focused only on just one disease entity. Data-driven approaches provide recommendations on an individualized level but require large amounts of empiric high quality data for reliable models and are often hard to interpret. Accessibility and availability of appropriate clinical data is rare today and will always be subject to challenges. Combining both approaches – knowledge-based and data-driven – has the potential to benefit from each advantage while overcoming limiting factors.

The CDSS described in this work targets the recommendation of an initial firstline (single-drug) pharmaceutical treatment for a given patient with diagnosed hypertension. Treatment options are one of the five main antihypertensive groups ACE inhibitor/ AT1- receptor blocker, beta blocker, calcium channel blocker and diuretics. The CDSS combines experience stored in empirical routine care documentation (data-driven) with evidence from literature (knowledge-based). The data-driven approach facilitates taking comorbidities, comedication, patient characteristics and living conditions of the target patient into account to provide more personalized recommendations than applying clinical guidelines only. Moreover, the proposed CDSS aims to recommend treatments in an explainable fashion to support SDM. We evaluate the proposed recommendation algorithm by benchmarking its output against recommendations of multiple specialist physicians using artificial test patients (case vignettes). Finally, we identify and discuss challenges and obstacles concerning the usage of routine care data as a basis for a data-driven antihypertensive treatment recommender system.

Method section describes the proposed CDSS prototype from a system architecture, algorithm, and user interface perspective, respectively. The routine care data (training data), the recommendations are based on, and the artificial test patients (test data) are detailed in System architecture section Finally, Treatment recommender algorithms section describes the evaluation strategies and results and Data-driven filtering and ranking section discusses and critically assess the results, findings and challenges.

## Methods

### System architecture

**Fig. 1:**
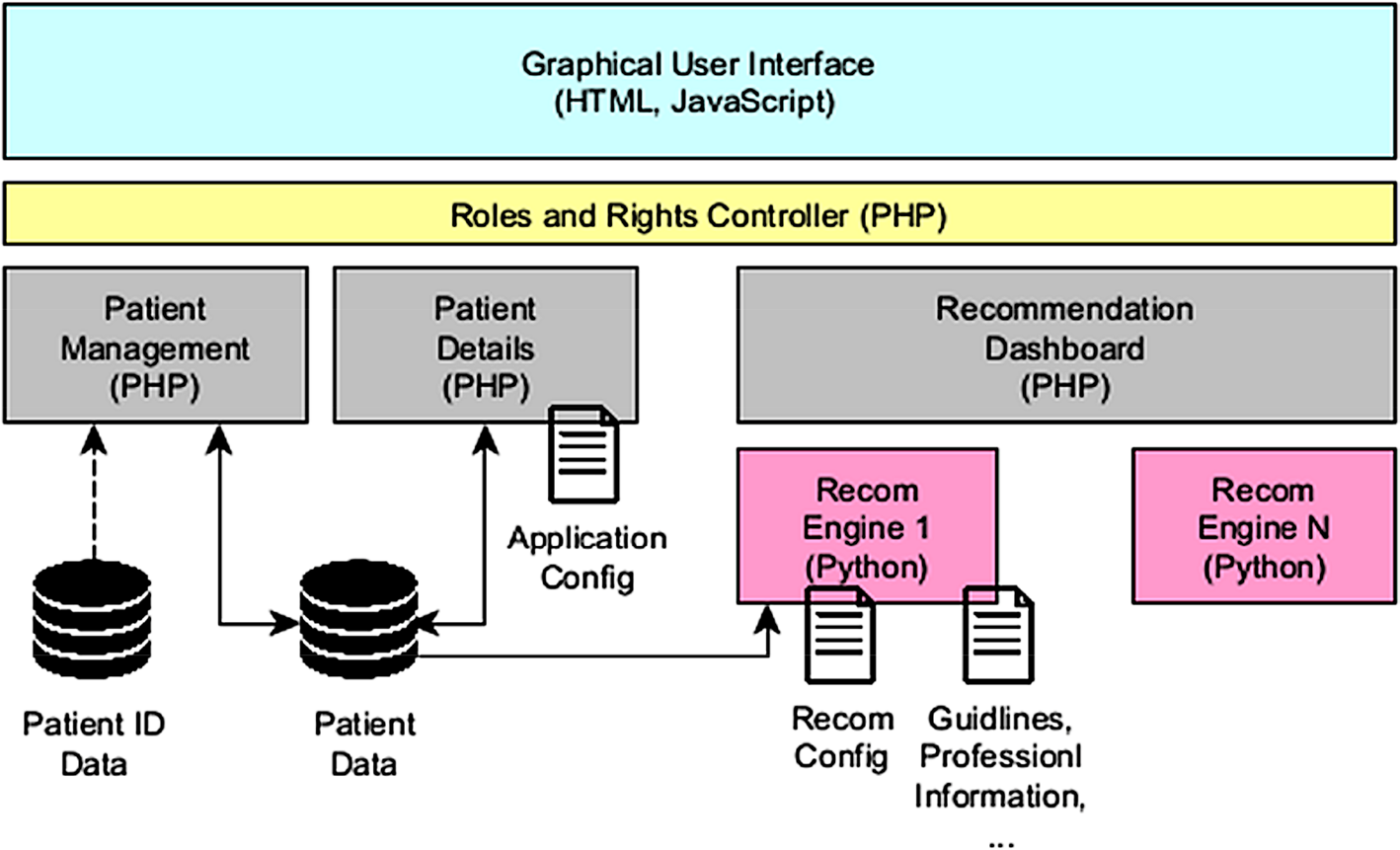
The treatment recommender system’s modular architecture

We have opted for a web application approach (client–server-model) and a browser-based graphical user interface (GUI). On the one hand, this approach ensures portability among platforms and devices (Desktop PC, Tablet, and Smartphone). On the other hand, the distributed architecture simplified maintenance and improvement and facilitates scalability by outsourcing of computing power and memory infrastructure. An architecture overview is given in Fig. 1.

Patient selection (Patient Management) and data input and presentation (Patient Details) is implemented in HTML using the Bootstrap Framework and PHP. To ease adaptation to evolving requirements and portability to other clinical applications, data input and presentation (Sect. *Patient data input and presentation*) is generated during runtime from an application configuration file (Application Config). The configuration file defines variables along with their source (here SQL database), data type, value range, grouping of variables and type of presentation (e. g. list, table, chart).

Independent recommendation engines can be included as services (micro service) written in Python using the web framework Flask. The recommendation dashboard (Sect. *Recommendation dashboard*), which is launched when selecting a recommendation engine, sends a recommendation request to the selected service. For this purpose, each recommendation engine provides a REST (REpresentational State Transfer) Application Programming Interface (API). In the request body, patient and visit identifiers are passed to the recommendation engine. Based on the passed identifiers, the respective service retrieves the required patient data from the database and computes the recommendations. The response body provided by the recommendation engine contains all recommendation data in a JSON (JavaScript Object Notation) string. Based on this data, the dynamic and interactive dashboard as described in Sect. *Recommendation dashboard* is generated. The actual data visualization is implemented in JavaScript using D3.js. Data input and representation including the patient database and each recommendation engine is containerized using individual Docker containers.

### Treatment recommender algorithms

#### Data-driven filtering and ranking

Recommender systems are widely applied in other domains, such as e-commerce, and subject of current research [11]. The recommendation algorithm proposed in this work is based on the identification of populations, i.e. subgroups, with similar characteristics as the target patient (patient pooling). Experience with treatment options within this population provide the basis for therapy recommendations. Within the context of recommender system research, such methods which rely on the experience and preferences of similar users fall in the category of neighborhood- or memory-based collaborative filtering [12]. In the context of clinical trial design, this approach can be interpreted as mimicking a personalized observational study. The neighborhood can be considered as a “personalized cohort”.

**Fig. 2:**
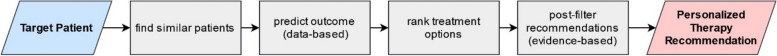
(1) Similar patients are filtered from the training patient database; (2) Observed treatments and outcomes are used to compute personalized outcome predictions; (3) Treatment options are ranked according to outcome predictions; The top-$\mathrm{N}$ ranked treatments are presented to the user; (4) Using evidence-based criteria, treatment options can optionally be post-filtered (Sect. *Knowledge-based labeling and post-filtering*)

The processing steps of the overall recommendation algorithm are shown in Fig. 2. The different recommendation engines implemented and evaluated within this work differ in the way (1) how similar patients are selected and (2) how treatment options are ranked and hence recommendations derived based on this neighborhood of similar patients.

Non-personalized recommendations are generated by including the entire training patient database as similar patients (RE-glob). This approach is considered a baseline method. In order to define a personalized neighborhood (RE-loc), the training patient database is filtered for similar patients based on predefined patient attributes $x$ and using the simple matching coefficient (SMC)

$${{\eta }_{j}=f}_{SMC}\left(x,{x}_{j}\right)=\frac{{\sum }_{k=1}^{K}s({x}_{k},{x}_{k,j})}{K}$$

A database patient $j\in J$ is included into a target patient’s neighborhood if ${\eta }_{j}$=1, i.e. it is similar regarding all $K$ attributes. The condition to be considered as similar, i.e. *matching*, however, depends on the attribute’s datatype, as detailed in the following.

In case of nominal or ordinal scaled attributes (e.g. gender) observations must be equal in order to be regarded as similar.

$$s({x}_{k},{x}_{k,j})=\left\{\begin{array}{c}1, x={x}_{k,j}\\ 0, x\ne {x}_{k,j}\end{array}\right.$$

In case of interval or ratio scaled attributes (e.g. age), a lower and an upper threshold define an interval around the target patient’s value of this attribute. To be considered as similar, a database patient must fall into this interval with the respective attribute’s value.

$$s({x}_{k},{x}_{k,j})=\left\{\begin{array}{c}1, {x}_{k,j}-{\Delta }_{k} \le x\le {x}_{k,j}+{\Delta }_{k}\\ 0, ({x}_{k,j}-{\Delta }_{k} >x)\wedge (x>{x}_{k,j}+{\Delta }_{k})\end{array}\right.$$

The attributes which define similarity and interval size of quantitative attributes are configured in the recommender engine’s settings. In the following, three patient attributes with the stated intervals are included: gender, age (+/-5 years) and body mass index (BMI (+/- kg/m^2)). This configuration is based on preliminary experiments.

In order to derive recommendations, therapy options $m\in M$ are ranked regarding an outcome prediction ${h}_{m}$ and the top-$N$ options are presented to the user. Outcome predictions ${\varvec{h}}\in {[0;1]}^{1\times M}$ are defined as

$${\varvec{h}}=\frac{{\varvec{\eta}}}{\Vert {\varvec{\eta}}\Vert }{\varvec{A}}$$

with $\Vert {\varvec{\eta}}\Vert$ being the ${\mathcal{l}}_{1}$-norm of the similarity vector ${\varvec{\eta}}\in {[0;1]}^{1\times J}$. Two prediction approaches are compared which differ in the way how the sparse patient-therapy-matrix ${\varvec{A}}\in {\{0;1\}}^{J\times M}$ is defined.

One basic approach (RE-pop) is to define ${\varvec{A}}$ as all treatments which were applied to patients in the patient database. This approach computes the distribution of occurrences of therapy options within a neighborhood and hence assumes the mode of the distribution to be the optimal option and solely relies on the physician’s choice. An alternative outcome-driven approach (RE-mean) defines $A$ as not only the application of treatments but also indicates whether the blood pressure endpoint 140/90 mmHg was reached or not. Hence, this approach estimates the chance to reach the blood pressure for a given treatment option by computing the ratio of similar patients reaching this endpoint. The predicted outcome can be interpreted as the probability to reach the blood pressure endpoint if treated with the given recommendation.

As outcome predictions can only be computed for treatment options which were applied at least once in the selected neighborhood, both approaches combine both, a selection of treatment options and a ranking based on predicted outcome.

Beyond the stated blood pressure endpoint, additional outcome dimensions can be extracted from the available data (see 4.1) and be considered depending on treatment focus. Summary statistics of these additional outcome indicators observed in the target patient’s neighborhood can be visualized as described in Sect. *Recommendation dashboard*. In case of nominal and ordinal scaled outcome measures (e.g. occurrence of adverse events), the distribution over treatment options is computed. In case of interval or ratio scaled attributes (e.g. change of systolic or diastolic blood pressure), the average outcome for each treatment option is computed and grouped into value ranges.

#### Knowledge-based labeling and post-filtering

For each of the target patients, the recommendation engine provides a personalized subset of treatment options which are ranked depending on the target patient’s characteristics. To facilitate evidence-based recommendations, treatments in the generated subset are labeled with information on contraindications, if available.

The underlying evidence-information about contraindications was manually extracted from the relevant clinical guideline [3] and the individual drugs’ professional information [13]. It was transferred into a computer processable structure and grouped by comorbidities, life situation and concomitant medication, as listed in table 1. For each identified contraindication the information listed in Table 2 was extracted from the information source.

**Table 1:** Considered patient characteristics as filter conditions for evidence-information on contraindications

Attribute	Description
Comorbidity	Additional confirmed diagnosis
Condition	Life situation, i.e. pregnancy, weight and age
Comedication	Drug interaction with antihypertensive medication

**Table 2:** Evidence-information on contraindications to label treatment recommendations with. Contraindication level can be used to filter treatment options from the recommendation list

Attribute	Description
Contraindication level	absolute, relative
Description	Description text (excerpt) from information source
Type	Comorbidity, life situation, drug interactions
Source type	Professional information, clinical guideline
Source	Source name and date

When generating recommendations, this information database is filtered conditional to the target patient’s characteristic, i.e. comorbidities, life situation and concomitant medication. The retrieved information is attached to the treatment options and shown to the user in the recommendation dashboard (Sect. *Recommendation dashboard*). In addition, contraindication levels (absolute, relative) can be used to hide, i.e. filter treatment options from the recommendation list (evidence-based post-filtering) as described in Sect. *Knowledge-based labeling and post-filtering*.

### Treatment recommender prototype

#### Roles and rights management

A role-based access control system was implemented to ensure only authorized persons have permission to access patient data (data protection) and particular functions depending on the user role (data security). Patient data can be read only by patients, patient data can be added and updated by physicians, and patient records can be deleted by administrators. User roles are combined hierarchically, meaning that higher-level roles (e.g. administrator) inherits permissions owned by all lower-level roles (e.g. patient and physician). Patients are only authorized to access their own data, physicians are authorized to access selected patients whereas administrators are able to access all patients. User roles and patient access are managed by administrators.

In this prototype, medical data (patient data) is stored pseudonymized. Patient identifying information, i.e. patient names, are stored in an independent database (Patient ID Data) at a third party trust authority. Pseudonymization facilitates access and modification of selected patient’s records.

#### Patient data input and presentation

After login, i.e. authorization, and depending on the user role, existing visits of one or several patients can be selected or deleted or new visits added. Moreover, new patients can be added or existing patients deleted.

**Fig. 3:**
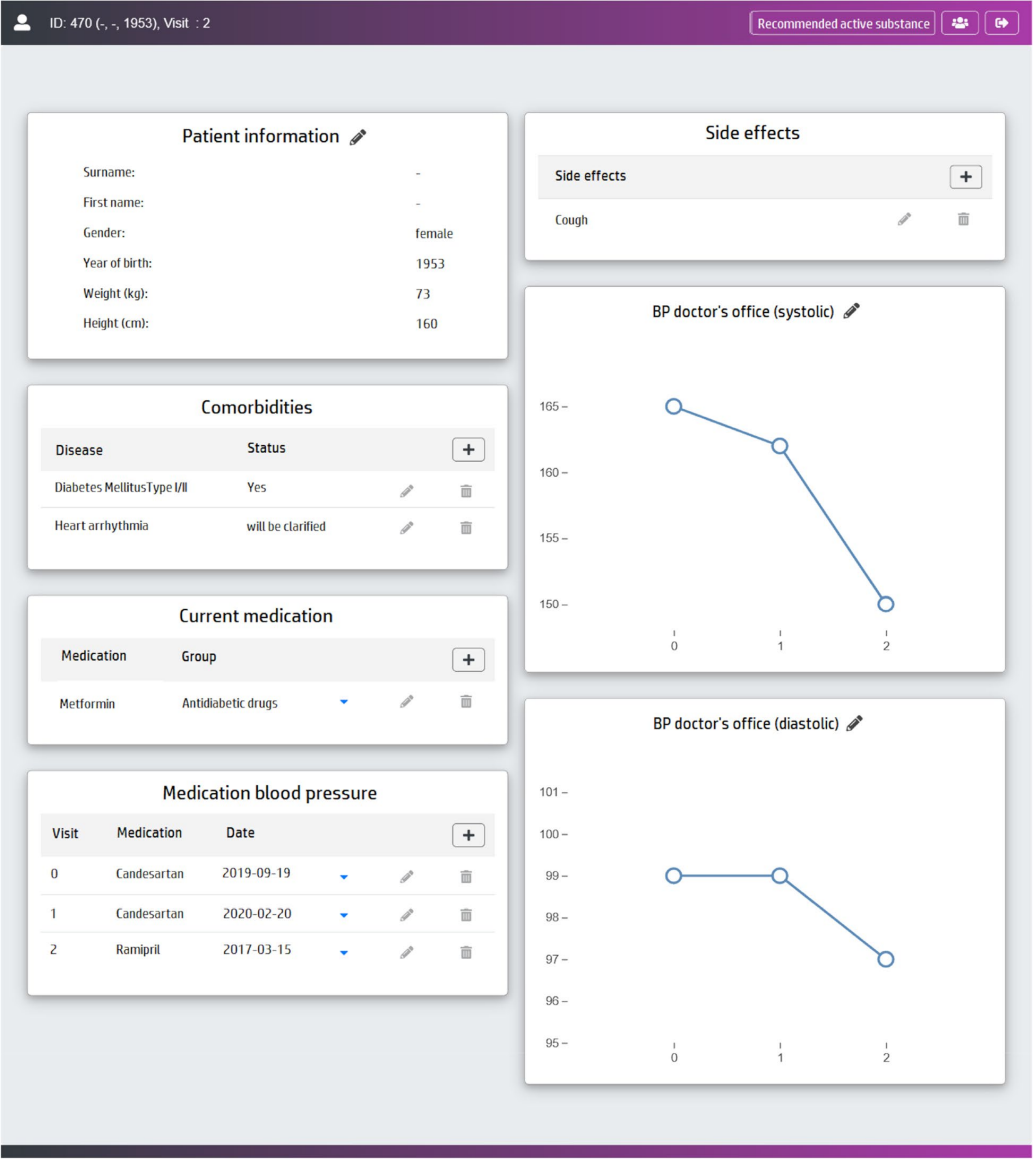
Patient data presentation and input. Patient data, such as demographic data, comorbidities, current medication, and blood pressure measurements, as well as information on previous medication and blood pressure are presented for the selected patient and consultation and are editable

Selecting an existing visit facilitates to show or edit patient data recorded for this visit. Data for each visit is organized in cards as shown in Fig. 3. Data is organized as a list of attributes (e.g. demographic data), tables (e.g. comorbidities) or charts (e.g. blood pressure readings).

If a new visit is created for an existing patient, the data from the previous visit is copied to reduce the input effort. In the following, the new visit is displayed and attributes can be adjusted if required.

#### Recommendation dashboard

**Fig. 4:**
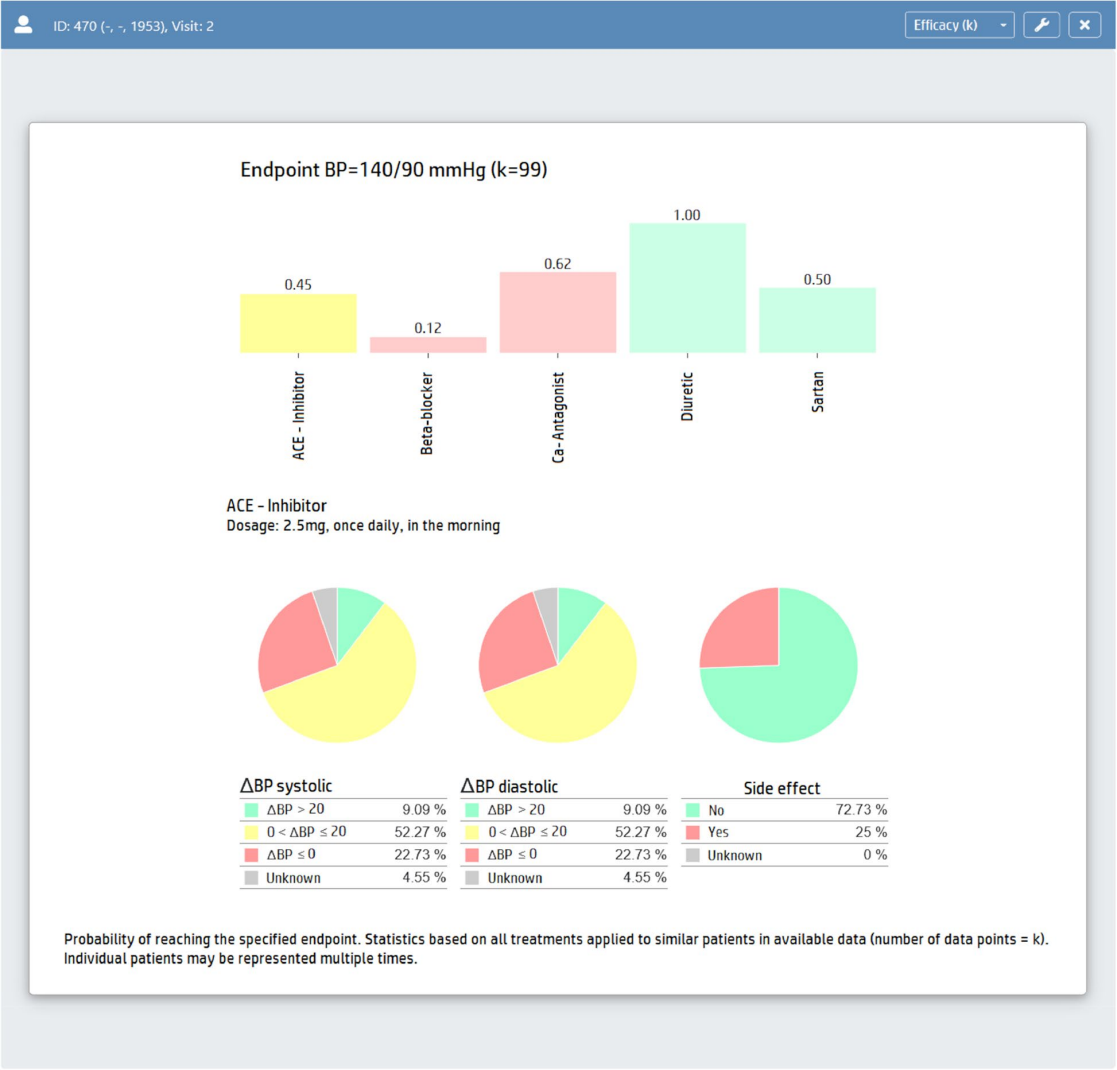
Therapy recommender dashboard. The endpoint estimates for each therapy option are visualized in a bar chart. The bar colors indicate no contraindications (green), relative (yellow), and absolutely (red) contraindication for the respective treatment option. By hovering over an option, summary statistics are shown for additional outcome parameters as pie charts (i.e. estimate of systolic and diastolic BP, probability for adverse events). Moreover, administration and dosage information are shown

The aim of the recommendation dashboard design is to provide (1) explainable recommendations and to (2) enable SDM. In addition, the dashboard provides evidence-based information regarding treatment options tailored to a specific patient and visit. From data presentation the therapy recommender dashboard can be launched by choosing a recommendation engine. Recommendations are created which relate to the currently selected visit. The upper bar chart in the recommendation dashboard (Fig. 4) visualizes all treatment options relevant for the target patient. Bar height visualizes the recommendation strength depending on the chosen recommendation engine, i.e. the popularity of treatments or outcome prediction (Sect. *Data-driven filtering and ranking*). Colors indicate absolute (red) and relative contraindication (yellow) of a therapy as derived from evidence-based rules (Sect. *Knowledge-based labeling and post-filtering*. Contraindicated options can optionally be hidden (filtered) from the chart and treatment options can be ordered by recommendation strength, alphabetically by name or price. Additionally, the current treatment is highlighted if the treatment target (140/90 mmHg) is reached for the target patient under this treatment to recommend its continuation. By hovering over a bar, administration and dosage information are shown. By clicking or tabbing on a bar, a popup window with additional information is shown: (a) Product names with price, (b) description of contraindication with information source, and (c) warnings with information source, if available (Sect. *Knowledge-based labeling and post-filtering*). The three pie charts visualize the estimated distribution of the additional outcome indicators change of blood pressure systolic and diastolic and side effects (Sect. *Data-driven filtering and ranking*). By hovering over a bar, the pie charts are updated with the values for this respective therapy option. By hovering over a fraction of a pie, the bar chart is updated with the distribution of the selected outcome parameter and value or value range, respectively. Suchlike, various aspects of treatment outcome can be included into the therapy decision. By visualizing interpretable estimates of various outcome dimensions, SDM can be facilitated which takes the patient’s preferences into account.

## Data

### Training data

The proposed recommendation engines are based on empiric routine-care data, i.e. real world evidence (RWE). The available data to represent real world evidence was extracted manually from health records, namely practice management systems from 7 general practitioners practices including 14 physicians treating patients with hypertension. The retrospective data collection included all patients with newly diagnosed hypertension from 2014 to 2020 in the specific health records. At the first doctor-patient contact (visit 0), the untreated blood pressure on the day of diagnosis was taken as baseline value. In subsequent doctor-patient contacts (V1-V4) blood pressure treated with antihypertensives was taken to retrieve an outcome measure. All blood pressure values combined with individual patient characteristics for each doctor-patient contact were transferred into a structured format.

Patients were enrolled in the study if (1) they agreed to participate in the study (informed consent), (2) a reliable hypertension diagnosis was available between 2014 and 2020, and (3) the diagnosed hypertension was untreated.

Two hundred ninety eight out of 5931 hypertension patients treated in the 7 practices met the inclusion criteria. In total, data from 900 doctor-patient contacts, i.e. visits, were extracted. Each patient is presented as a set of basic information, namely patient ID, gender and date of birth. Each patient visit is represented as visit ID, anthropometric data, diagnosed comorbidities, living situation (e.g. pregnancy, breastfeeding), concomitant medication, current blood pressure values, prescribed antihypertensive medication and adverse drug events. As discussed in Sect. *Treatment recommender algorithms*., only a fraction of this information is actually used by the recommendation algorithm. From a longitudinal sequence of patient visits, blood pressure and adverse drug events can be associated with the antihypertensive medication prescribed in a previous visit. Based on the blood pressure curve, three outcome parameters are extracted to assess the antihypertensive treatment: change in (i) systolic and (ii) diastolic blood pressure due to treatment and the (iii) dichotomous endpoint whether the blood pressure endpoints 140/90 mmHg is reached.

Data points are extracted from the longitudinal sequence of applied treatment and associated outcomes to serve as training data for the recommendation engines described above. From each patient’s visits the contiguous subsequence of visits in which a specific treatment was performed is extracted. Thus, the number of datapoints corresponds to the number of contiguous subsequences of treatments across all patients. If a patient's treatment has been changed, the treatment before the change affects the observed outcome. In clinical trials, this effect is typically eliminated by washout periods. Here, only the initial treatment is considered as a data point in order to be able to reliably assign the outcome with a specific treatment. This reduces the total number of data points to the number of patients, i.e. 278.

### Test data

Our recommendation algorithm is evaluated using artificial test patients, i. e. case vignettes. Vignette analysis is a survey experiment in which respondents, i.e. clinical experts, are confronted with hypothetical patients and cases. Vignette analysis is a suitable method for illustrating the reality of life and assessing a person's local circumstances [14]. A total of 23 vignettes are created to evaluate the proposed recommender system compared to the performance of general practitioners. The case vignettes contain classic cases formulated by an expert according to guidelines and assessed as realistic by general practitioners. They contain the following information, which is, however, only partially used by the algorithm: 24-h blood pressure measurement, age, smoking status, gender, height, weight, secondary diseases, abnormal laboratory findings (HbA1c, blood lipid values, etc.) and existing long-term medication.

Based on the information given, five physicians are asked to recommend one appropriate first-line antihypertensive treatment (agent) for each of the 23 cases. All five physicians are general practitioners with years of experience treating hypertension patients.

## Evaluation and Results

### Evaluation strategy

Since the focus is on recommending the potentially most successful treatment rather than mimicking the physicians’ decision, only the outcome-driven approach (RE-mean) is used for evaluation. The recommender engine’s settings, i.e. the attributes to be considered, are configured as described in Sect. *Data-driven filtering and ranking*. This personalized approach is compared with the non-personalized baseline recommendation (RE-glob). Moreover, to assess the impact of evidence-based post-filtering (Sect. *Knowledge-based labeling and post-filtering*), two variants of each approach are included into the analysis: data-driven only (RE-glob, RE-loc) and including post-filtering (RE-glob-eb, RE-loc-eb). Here, all absolute contraindications due to comorbidities, life situations or drug interactions are filtered regardless of the underlying evidence source (professional information, clinical guideline). A total of four variations are compared (Table 2).

The performance of the recommender system was evaluated using the case vignettes described in Sect. *Test data*. The generated recommendations are compared with the recommendations of the five physicians $p$. Hence, for each case vignette $i\in I$ five ground truth treatments are available represented as a one-hot-encoded vectors ${{\varvec{t}}}_{i,p}\in {\{0, 1\}}^{1\times M}$. The recommendation engine outputs a vector of treatment options along with the associated outcome predictions ${{\varvec{h}}}_{i}$. Recommendation list entries, i.e. treatment options, with the top-$N$ ($N$=1, 2 and 3) largest outcome prediction values are encoded as one and zero otherwise, resulting in ${{\varvec{h}}}_{i}^{0}$. Hence, agreement ${r}_{i,p}\in \{$ 0, 1} between recommender system output and physician $p$ for case $i$ is computed as

$${r}_{i,p}={{{\varvec{h}}}_{i}^{0}{\varvec{t}}}_{i,p}^{T}$$

and averaged over all case vignettes, yielding ${r}_{p}\in [\mathrm{0,1}]$.

The average agreement ${r}_{all}$ (agree_all) with all experts $p$ is computed from ${r}_{p}$. Moreover, agreement with any expert ${r}_{any}$ (agree_any) and the agreement with the majority vote of all experts ${r}_{majority}$ (agree_majority) is computed. Here, ground truth vectors ${{\varvec{t}}}_{{\varvec{i}}}\in {\{0, 1\}}^{1\times M}$ encode the elementwise conjunction of ${{\varvec{t}}}_{i,p}$ over all $p$ or the majority votes over all $p$, respectively.

### Evaluation results

**Fig. 5:**
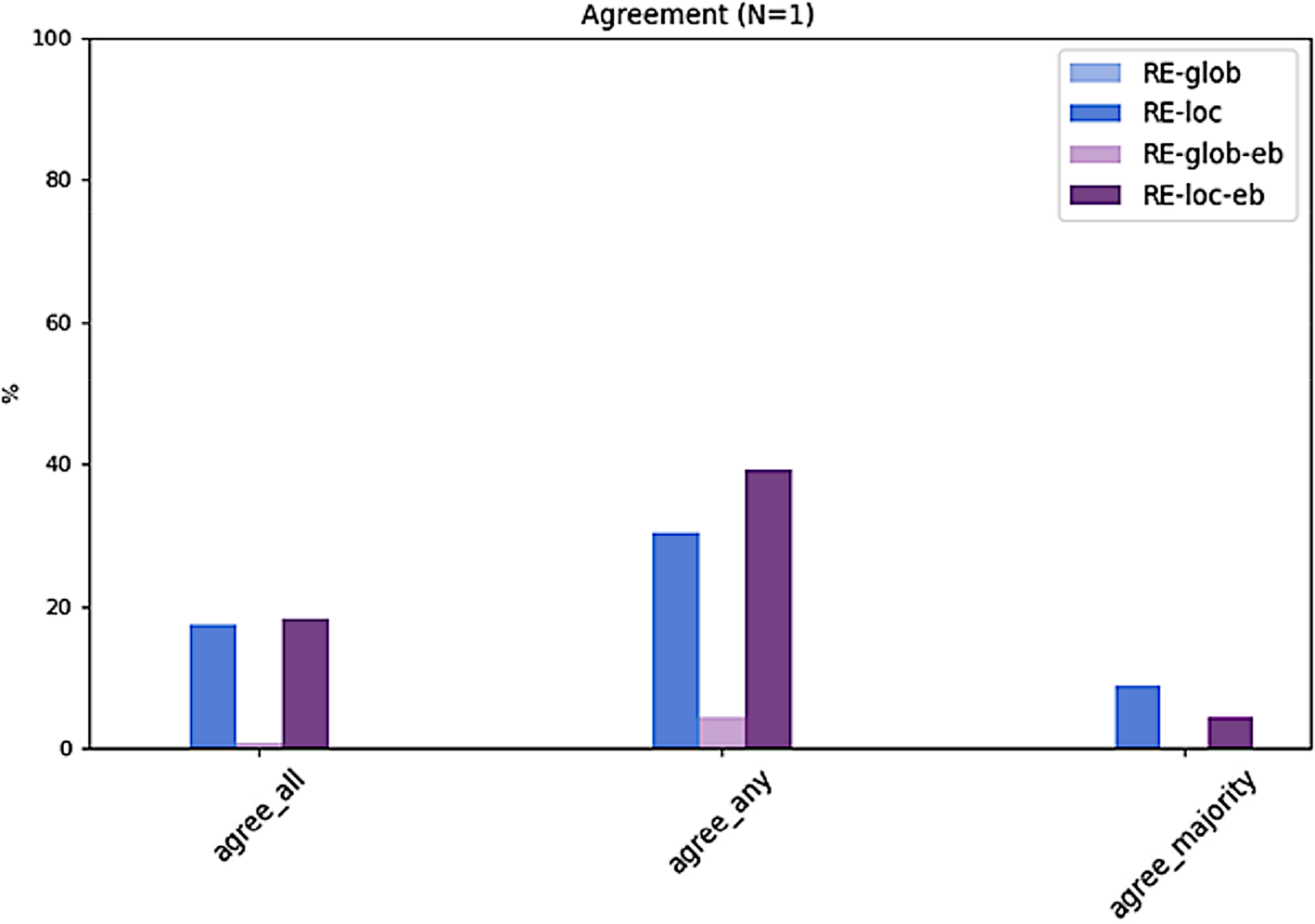
For each algorithm, agreement with all experts (agree_all), with any expert (agree_any), and with the majority vote of all experts (agree_majority) for the top-1 ranked treatment recommendations

**Fig. 6:**
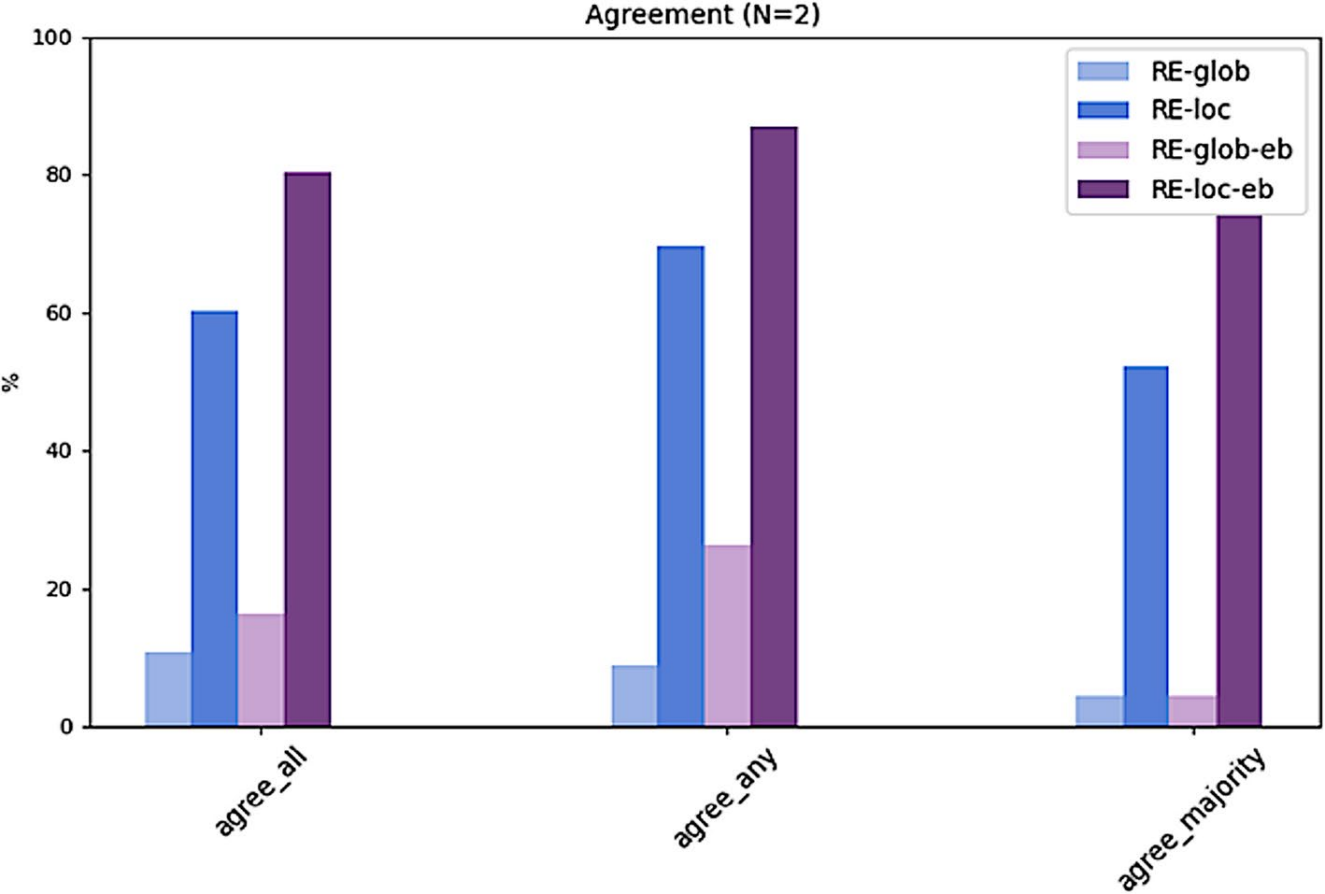
For each algorithm, agreement with all experts (agree_all), with any expert (agree_any), and with the majority vote of all experts (agree_majority) for the top-2 ranked treatment recommendations

**Fig. 7:**
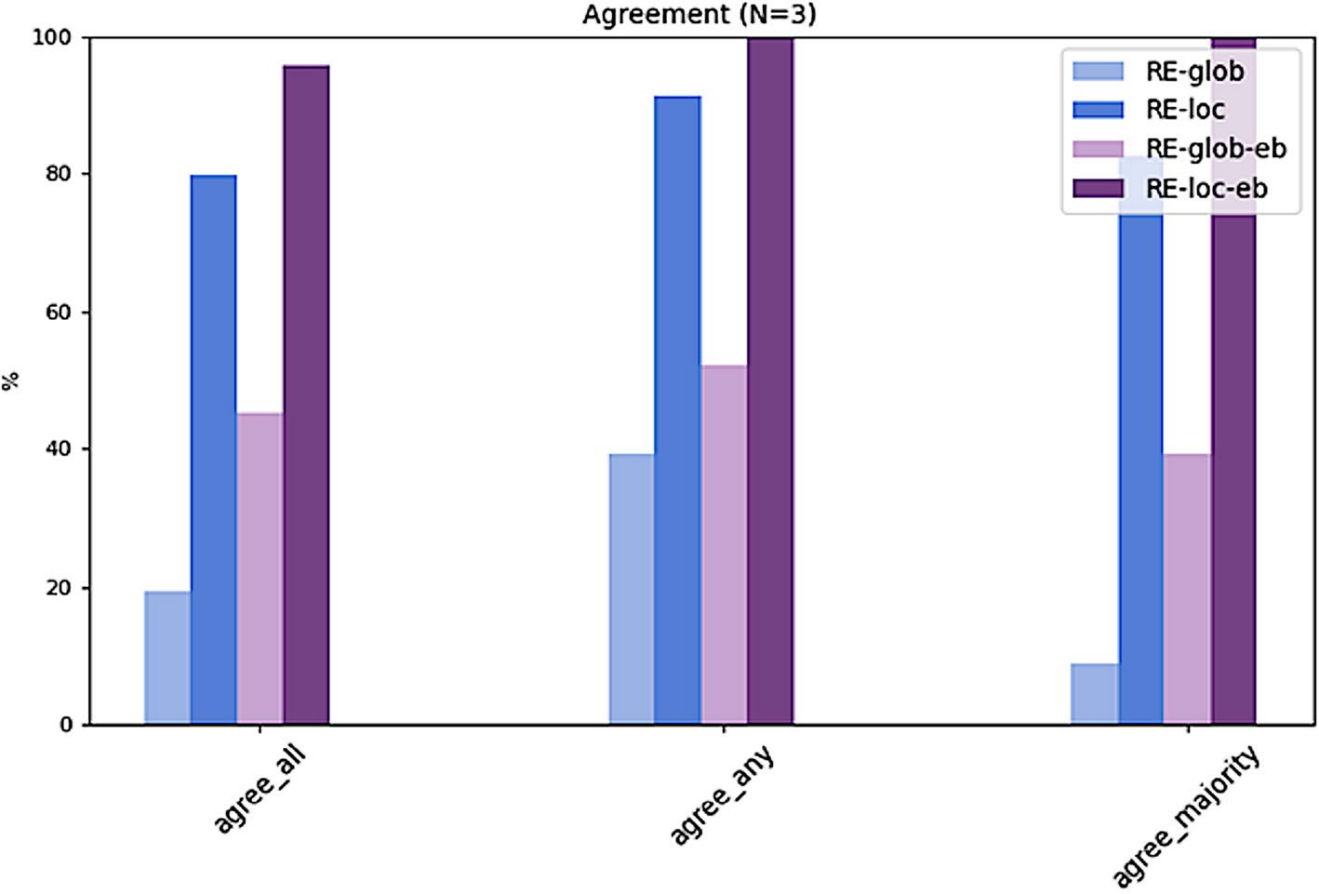
For each algorithm, agreement with all experts (agree_all), with any expert (agree_any), and with the majority vote of all experts (agree_majority) for the top-3 ranked treatment recommendations

The results presented in Table 3 and Figs. 5, 6 and 7 clearly show that all top-N recommendation lists benefit from a selected local neighborhood which recommendations are based on. The local, i.e. personalized approaches (RE-loc, RE-loc-eb) clearly outperform the global approaches (RE-glob, RE-glob-eb). Hence, the algorithm with the selected configuration is capable of identifying a meaningful neighborhood for a given target patient. As shown in preliminary experiments, RE-loc and RE-loc-eb benefit from small neighborhoods. The configuration (age+/-5 years, gender) which determines the neighborhood results on average in 28.35 (16.44) similar cases. For larger age value ranges the results deteriorate.

**Table 3:** Summary of results. For each algorithm, agreement with all experts (agree_all), with any expert (agree_any), and with the majority vote of all experts (agree_majority) is shown for the top-1, top-2, and top-3 ranked treatment recommendations

**RE-glob**	top-1	0.00 (0.00)	0.00	0.00
	top-2	0.11 (0.08)	0.09	0.04
	top-3	0.19 (0.18)	0.39	0.09
**RE-loc**	top-1	0.18 (0.10)	0.30	0.09
	top-2	0.60 (0.06)	0.70	0.52
	top-3	0.80 (0.07)	0.91	0.83
**RE-glob-eb**	top-1	0.01 (0.02)	0.04	0.00
	top-2	0.16 (0.14)	0.26	0.04
	top-3	0.45 (0.14)	0.52	0.39
**RE-loc-eb**	top-1	0.18 (0.14)	0.39	0.04
	top-2	0.80 (0.05)	0.87	0.74
	top-3	0.96 (0.03)	1.00	1.00
		**agree_all**	**agree_any**	**agree_majority**

Moreover, performance of the solely data-based approaches (RE-glob, RE-loc) is improved by applying the described evidence-based post-filtering rules (RE-glob-eb, RE-loc-eb). This is particularly evident for the top-2 and top-3 recommendations (Figs. 6 and 7). The local approach with evidence-based exclusion rules (RE-loc-eb) is able to rank the given treatment options such as that – averaged over all five ground truths – in 96% of the test cases the expert recommendations are among the top-3 algorithm recommendations (agree_all). In 100% of the cases the top-3 recommendations agree with any of the experts (agree_any) and with the experts' majority vote (agree_majority).

**Fig. 8:**
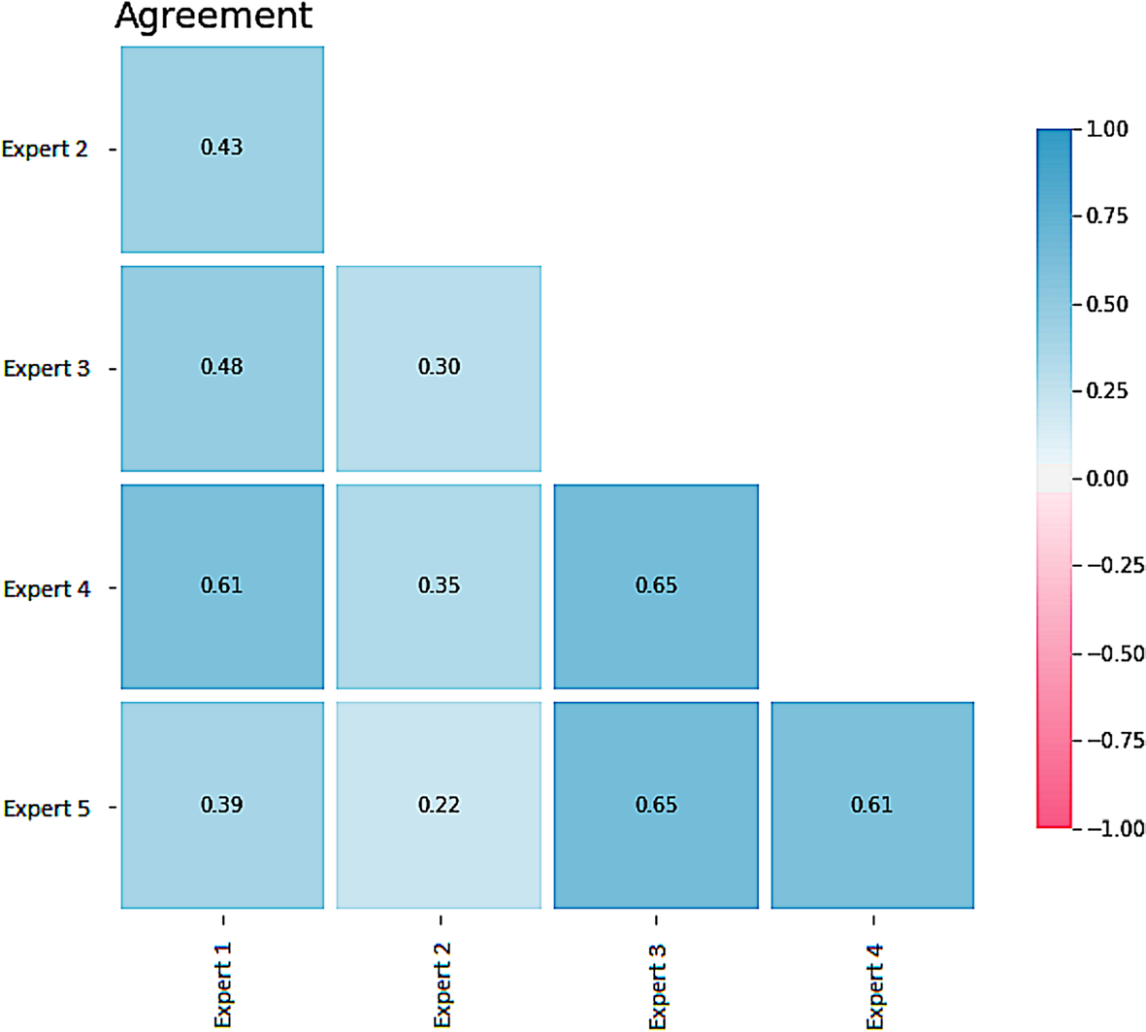
Inter-rater agreement among experts

Comparing the achieved results (agree_all) with the inter-rater agreement among experts (Fig. 8), the top-2 and top-3 recommendations of the local evidence-based approach (RE-loc-eb) on average agree even more often with the experts than the experts among each other.

## Discussion

### Interpretation of the results

As the study showed, the prototype presented is capable of providing personalized recommendations for a specific patient with a large agreement compared to well-trained general practitioners. Exploiting experience from a subgroup of similar cases is particularly beneficial in contrast to recommendations based on average outcome. Hence, personalizing recommendations benefits the quality of treatment recommendations. Prerequisite is the selection of a meaningful subgroup, i.e. neighborhood.

The advantage of the presented outcome-driven neighborhood-based approach is twofold: the algorithm (1) selects a subset of suitable treatment options and (2) ranks them according to the average outcome observed in the selected neighborhood. This approach can provide additional interpretability of the recommendation if the considered data subset is visualized.

The recommendation engine is not only based on a data-driven component, but also takes clinical evidence into account. This hybrid approach further improves the quality of the recommendation.

Nevertheless, it must be kept in mind that recommendations are outcome-driven, but the agreement between algorithms and expert recommendations is measured as evaluation metric. The underlying outcome prediction cannot be assessed because the given test data (case vignettes) don't provide ground truth for an actually observed outcome. In addition, it is very likely that the physicians' decisions (ground truth) are also influenced to a greater extent by the costs of medication. ACE inhibitors are by far the cheapest option. As the treatment recommendation system presented is exclusively outcome-driven, costs are not taken into account.

### Limitations

The major limitations of this work are quality and sample size of the training data and the small size of the test data on which the results are based. This not only limits the method’s performance but also the generalizability of the results demonstrated. A larger training data set containing more unique patients also has the potential to take advantage of more sophisticated algorithms from machine learning research, automatic features selection, or metric learning [15].

Furthermore, data quality is insufficient. Routine care documentation in medical practice management software (PMS) or hospital information systems (HIS) is not intended for scientific purposes. Hence, using such documentation as training data source is associated with multiple obstacles: (i) documentation is unstructured and non-standardized, (ii) often incomplete and (iii) lacking open standards require manual and error-prone data extraction. Especially information on lifestyle changes, quality of life, or medication adherence but also therapy and dosage changes were often insufficiently documented in the existing data (patient records). Standardization and semantic interoperability of data sources and systems is a key requirement to facilitate automatic data processing of medical data in general.

Evidence-based rules were extracted and structured manually, as described in Sect. *Knowledge-based labeling and post-filtering*. Keeping such rules up-to-date and consistent is a laborious task. Current results from clinical trials and up-to-date information from pharmacovigilance can hardly be processed automatically today, but information must be extracted and evaluated manually by human experts. But even machine-processable clinical guidelines, best practices, or clinical pathways are not publicly available today and there are no standards for computerization of guidelines. Standardization of medical knowledge, however, is essential to establish a learning health system [16].

### Future works and perspectives

As an alternative data source, existing structured databases, such as patient registries or clinical data repositories (CDR) [17], must be interfaced in future works. For this, challenges regarding syntactic and semantic interoperability must be handled and obstacles regarding data protection regulations must be overcome [18]. However, also unstructured health record data can be leveraged in future works by applying natural language processing (NLP) techniques [19].

In terms of real world application of such systems in everyday clinical practice, usage and acceptance will depend on two main issues: (i) integration into existing systems and workflows and (ii) trust. Whereas the first particularly concerns interoperability and usability, the latter requires proof of benefit through large-scale studies. Obstacles concerning interoperability will potentially change in the near future in Germany, driven by governmental initiatives to digitize health, such as introduction of the electronic patient record (ePA), advanced usage of standardized terminologies  (e.g. SNOMED-CT [20]) and increasing popularity and application of data exchange standards such as FHIR [21]. Future work will therefore concentrate on evaluating the system in a more exhaustive setting while enhancing integration into existing systems, e.g. PMS or HIS.

Requirements concerning usability are easy and intuitive interaction and accessibility of information and recommendations. For routine care application, recommendations have to be provided instantly without long delays at the point of care, where decisions are made [10]. Usability and user experience will definitely foster the acceptance of such systems. Future work will therefore also focus on (i) evaluation of the proposed therapy recommender system's user interface in terms of usability and user experience and (ii) the proposal of alternative user interfaces such as e.g. voice control.

To assess the outcome and success of hypertension treatment, this study only considers blood pressure and adverse events. Additional outcomes such as patient reported outcome measures (PROMs), e.g. quality of life, remain unconsidered in the current version and are also subject of future research.

## Conclusion

In this work we proposed a medication recommendation system and presented a prototype for the treatment of hypertension. To exploit advantages, remedy drawbacks, and promote acceptance, we proposed a hybrid system combining both rule-based and data-driven recommendations. The prototype was evaluated in a small study using artificial test patients (case vignettes) and compared with clinical experts. Overall, the feasibility and performance of such a system was demonstrated. The major challenges in developing such a CDSS arise from a lack of availability of suitable training and evaluation data and absence of standardized medical knowledge such as computerized guidelines.

If these challenges are solved, CDSSs as the demonstrated treatment recommender system can support physicians with exploiting knowledge stored in routine care data, help to comply with the best available clinical evidence and increase patient adherence by reducing side-effects and individualizing therapies. Such systems will play an important role in future healthcare. This work aims to contribute to fundamental ideas in this field.

## Data Availability

The datasets generated and/or analysed during the current study are not publicly available due to reasons of sensitivity but are available from the corresponding author on reasonable request. The same applies to the software source code.

## Abbreviations

API: Application Programming Interface
BP: Blood Pressure
CDR: Clinical Data Repository
CDSS: Clinical Decision Support System
DEGAM: German College of General Practitioners and Family Physicians
EbM: Evidence-based Medicine
ESC: European Society of Cardiology
GUI: Graphical User Interface
HIS: Hospital Information System
JSON: JavaScript Object Notation
NLP: Natural Language Processing
PMS: Practice Management System
PROMs: Patient Reported Outcome Measures
RWE: Real World Evidence
REST: REpresentational State Transfer
SDM: Shared Decision-Making
